# Transcriptomic analysis of three fig (*Ficus carica* L.) genotypes reveals metabolic reprogramming during seasonal development

**DOI:** 10.3389/fpls.2026.1866576

**Published:** 2026-07-02

**Authors:** Pasqualina Colasuonno, Ilaria Marcotuli, Bachir Balech, Monica Santamaria, Andrea Mazzeo, Stefania Lucia Giove, Giuseppe Ferrara, Agata Gadaleta

**Affiliations:** 1 Department of Soil, Plant and Food Sciences, University of Bari ‘Aldo Moro’, Bari, Italy; 2 Institute of Biomembranes, Bioenergetics and Molecular Biotechnologies, Consiglio Nazionale delle Ricerche, Bari, Italy

**Keywords:** brassinosteroid biosynthesis, caprifig, main crop, metabolic shift, photosynthesis-antenna proteins, seasonal transcriptomics, syconium development, TCA cycle

## Abstract

Seasonal development in perennial fruit species involves extensive metabolic modifications that support growth and environmental adaptation, but the molecular basis of coordination between primary and secondary metabolism in fig (*Ficus carica* L.) remains poorly characterized. To elucidate the seasonal regulation of metabolic pathways during syconium development, transcriptomic analyses were conducted on three fig genotypes (a caprifig, and two cultivars Dottato, and Petrelli) representing distinct reproductive types, sampled at two critical phenological stages (April and July) corresponding to breba growth and main crop development, respectively. Stringent pathway analysis of previously generated RNA-seq datasets highlighted a consistent metabolic reprogramming associated with both phenological stage and genotype differences, with pronounced modulation of biochemical pathways. Pathway filtering identified 15 pathways (45 unique genes) mainly associated with secondary metabolism and biosynthetic processes that showed higher expression in April (considered as up-regulated genes), and 23 pathways (79 unique genes) principally related to primary metabolism including photosynthesis and respiration that showed higher expression in July (considered as down-regulated genes). Among the pathways showing higher expression in July, photosynthesis-antenna proteins (map00196) and the tricarboxylic acid (TCA) cycle (map00020) exhibited the most consistent seasonal changes, with gene-level expression increasing 1.5- to 2.9-fold from April to July. A strong positive correlation (*r* = 0.77) between these pathways revealed tight coordination between light-harvesting capacity and central respiratory metabolism. Among the pathways showing higher expression in April, glucosinolate-, brassinosteroid-, and nucleotide-sugar-related processes were more highly expressed in April, while riboflavin metabolism increased in July, reflecting a shift from defense and growth functions in spring to energy-intensive metabolism during summer syconium development. Seasonal signals applied stronger and more uniform regulatory effects than genotypic or sex-related differences, though cultivar-specific metabolic characteristics were observed. Dottato exhibited the highest photosynthetic and respiratory gene expression in July, consistent with its robust parthenocarpic syconium development and ripening, while Caprifig displayed elevated TCA cycle activity potentially supporting specialized pollen production. These results revealed a fundamental metabolic architecture coordinating energy acquisition, respiratory activity, and biosynthetic processes during seasonal syconium development. The identified transcriptional networks provide molecular targets for breeding programs aimed at improving fig cultivation under changing environmental conditions.

## 1 Introduction

The common fig (*Ficus carica* L.) is a perennial fruit tree with a long history of cultivation and domestication, particularly across the Mediterranean regions where it represents both an economically and culturally significant crop (Ben Temessek et al., 2023). Its prevalence in this area is largely attributed to its tolerance to drought and high temperatures, along with the nutritional and functional properties of its fruits (Mazzeo et al., 2024). The common fig is characterized by a specialized urn-shaped inflorescence known as a syconium (commonly referred to as the fruit), an inverted fleshy receptacle that houses hundreds of unisexual flowers, whose true botanical fruits are the small drupelets located inside this structure. *F. carica* belongs to the genus *Ficus*, the largest within the *Moraceae* family, which involves a wide diversity of morphological forms and reproductive strategies (Berg and Corner, 2005; Chawla et al., 2012). Beyond its agronomic relevance, the common fig displays complex reproductive biology and marked phenological plasticity (Chmarkhi et al., 2025). Fruit quality and yield depend strongly on cultivar choice and on key processes such as bud activation and differentiation (Marcotuli et al., 2020; Ma et al., 2020).

Fig reproductive biology is characterized by remarkable diversity among cultivar types, with different degrees of pollination dependence and fruiting patterns. Fig development involves complex transcriptional reprogramming across distinct phenological stages that coordinates vegetative growth, reproductive transitions, and metabolic modifications in response to environmental signals (Marcotuli et al., 2023a). This regulatory flexibility is particularly relevant in fig due to its capacity to undergo multiple fruiting cycles and a wide range of developmental behaviors observed among cultivars (Singh et al., 2023).

Bud development and differentiation are central events influencing productivity and derived from the interaction between internal and external factors, including temperature and photoperiod (Flaishman et al., 2008). Ma et al. (2020) reported a direct genetic link between meristem activity and syconium production by identifying candidate regulators of flower bud differentiation, including the *LEAFY* homolog (*FcLFY*) gene. In parallel, *RAN1* (Responsive-To-Antagonist1) and *AGAMOUS* homolog genes have emerged as candidate regulators of flowering and sexual differentiation in fig (Mori et al., 2017), highlighting the dynamic and context-dependent nature of reproductive control in this species.

Elucidating the molecular mechanisms underlying these processes requires integrative approaches linking primary metabolism (energy source) with secondary metabolic pathways involved in stress responses and syconium quality determination (Bao et al., 2023; Wang et al., 2024). Metabolic coordination across developmental stages is particularly critical in fig, where distinct fruiting phases and types (breba and main crop or even late crop) may impose different energetic and biosynthetic demands on the plant. Recent genomic and transcriptomic resources for *F. carica*, including haplotype-resolved genome assemblies (Usai et al., 2025), integrated metabolomic studies (Sun et al., 2025), and gene-expression analyses across developmental stages and environmental contexts in several fruit crops (Vishwanath et al., 2025; Yuan et al., 2025), have provided valuable tools for molecular investigations.

In fig, however, most studies have targeted individual traits or specific stress conditions, leaving a limited understanding of how biochemical networks are orchestrated over the growing season. Although seasonal changes in photosynthetic capacity and leaf carbohydrate dynamics have been physiologically characterized (Vemmos et al., 2013), the transcriptional mechanisms underlying these responses remain largely unexplored. Comparative transcriptomic analyses across cultivars and phenological phases therefore represent a powerful strategy to uncover regulatory steps and metabolic transitions associated with developmental flexibility.

In this study, a comprehensive RNA-seq analysis was conducted on three fig genotypes (cvs. Dottato, Petrelli, and a local caprifig) representing distinct reproductive types at two key phenological stages (April and July) to investigate the transcriptional profiles of seasonal metabolic coordination. The objectives were to identify differentially expressed genes (DEGs) associated with primary and secondary metabolic pathways across genotypes and developmental stages, and to elucidate coordinated transcriptional responses associated with cultivar-specific reproductive behaviors. Particular attention was given to the potential functional coupling between photosynthesis-related pathways and respiratory metabolism during syconium development. The results provide novel insights into the transcriptional networks integrating metabolic reprogramming, seasonal regulation, and reproductive development in fig, offering a comparative molecular framework for understanding phenological diversity in this species.

## 2 Materials and methods

### 2.1 Plant material

**Figure 1:**
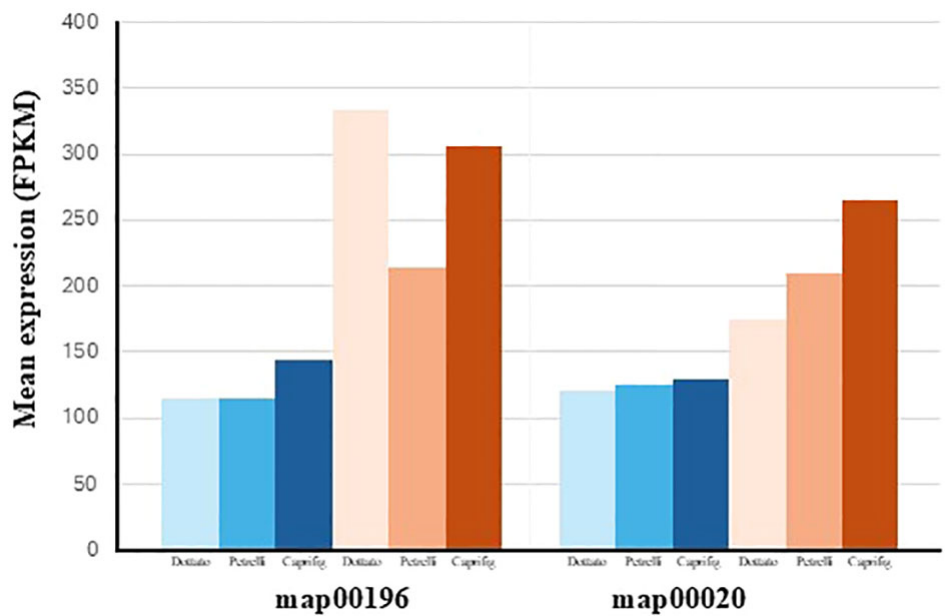
Differential pathway response (map00196 and map00020) in Petrelli, Dottato and Caprifig figs between April (blue colors) and July (yellow colors).

**Figure 2:**
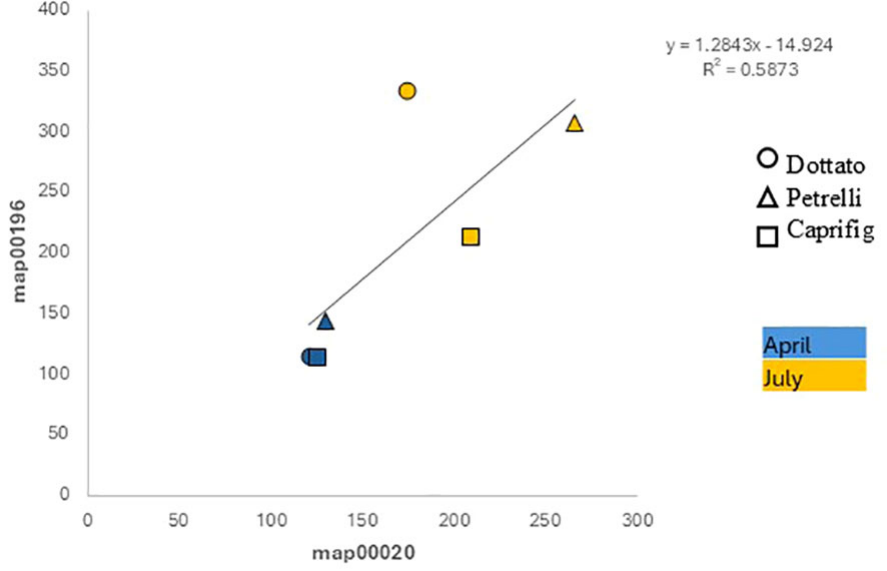
Correlation between central energy metabolism (map00020) and photosynthetic antenna proteins (map00196) across fig cultivars and seasons (April in blue symbols, July in yellow colours).

The three fig genotypes analyzed in this study are grown at the Educational and Experimental Station “P. Martucci” belonging to the University of Bari “Aldo Moro”, located in the countryside of Valenzano (Bari province, Italy). The genotypes include two female edible fig cultivars and one male caprifig. Dottato is a common-type female cultivar characterized by parthenocarpic fruit development and the production of a single main crop during summer, with only occasional breba formation. Petrelli is a San Pedro-type female cultivar (Ferrara et al., 2016) that produces breba fruits on one-year-old shoots in spring and may develop a reduced main crop during summer when pollination occurs from caprifig. The local caprifig represents the male form of *Ficus* carica and produces pollen-bearing syconia (profichi) in spring-early summer, which host *Blastophaga psenes* wasps that, upon emergence, carry pollen to female edible fig syconia (caprification). During summer-autumn, the caprifig produces mammoni fruits (few), followed by overwintering mamme fruits. Plant material and sampling procedures were conducted as follow: syconia buds were sampled from open-field trees managed according to conventional Mediterranean horticultural practices adopted for fig orchards. Collections were performed at two representative phenological time points: April and July, representing two ecologically relevant and contrasting seasonal developmental phases associated with early reproductive development and advanced fruit growth, respectively. Although the three genotypes differ in reproductive behavior and fruiting habit, these sampling dates were selected to represent comparable seasonal developmental windows rather than strictly identical phenological stages. For each genotype and sampling date, three independent biological replicates (three plants) were considered (total 9 plants sampled), one replicate for each time sample was made by a pool of 8 syconia buds, rapidly snap-frozen in liquid nitrogen, and preserved at -80°C prior to RNA isolation. Representative photographs of the syconia at both sampling time points are provided in **Figures 1**, **2**.

### 2.2 RNA extraction, library preparation, and sequencing workflow

Total RNA was isolated from syconium buds using the RNeasy Plant Mini Kit (Qiagen). For each genotype and sampling date, three independent biological replicates were used, each with three technical replicates; considering that non-significant variation was detected among technical replicates, the mean was used for subsequent analysis. RNA quality and quantity were assessed using a NanoDrop 2000 (Thermo Scientific) and checked on 1.5% agarose gel. RNA integrity was evaluated with Bioanalyzer 2100 and TapeStation 4200; only samples with a RIN higher than 8 were used for sequencing. Sequencing libraries were prepared using the TruSeq RNA Sample Preparation Kit v2 (Illumina) and paired-end sequencing (2 × 100 bp) was performed on an Illumina HiSeq 2000 platform. Raw reads were quality-filtered and adapter-trimmed using Trimmomatic v0.36 (Phred score < 20). Clean reads were mapped to the *F. carica* draft genome assembly (GCA_002002945.1) using Bowtie2, followed by *de novo* transcriptome assembly with Trinity. Functional annotation was performed using the Trinotate pipeline, and differential expression analysis was conducted with edgeR (|log_2_FC| ≥ 1.5, FDR < 0.05), this threshold is more stringent than the conventional |log_2_FC| ≥ 1, it was deliberately chosen to reduce false positives and focus on the most robustly modulated pathways.

### 2.3 Functional annotation of DEGs

Amino acid sequences of DEGs assigned to at least one GO term were searched against a local copy of KEGG orthologous (KO) hidden Markov model (HMM) profiles using the *hmmsearch* algorithm (HMMer 3.4; http://hmmer.org/). Each sequence was assigned to a specific KEGG pathway through the association of matched KO profiles. Although initial annotation was based on available *Ficus carica* genomic resources, comparative annotation against well-characterized model and related plant species was additionally performed to improve functional assignment reliability and pathway interpretation, given the still limited functional annotation available for fig. Pathway names were retrieved using the Application Programming Interface (API) provided by the KEGG database (Kanehisa et al., 2025). The searched *Ficus carica* DEG sequences were grouped by KEGG pathways to prioritize their functional profile. The same pathways were also confirmed in gene annotations from model and closely related plant species, including *Arabidopsis thaliana*, *Morus notabilis*, *Vitis vinifera*, and *Populus trichocarpa*, and were recorded accordingly for subsequent comparative analyses. It should be noted that the terms “up-regulated” and “down-regulated” refer to the overall pathway-level enrichment direction in the seasonal comparison (July vs April): pathways classified as down-regulated are those enriched in genes with higher expression in April, while up-regulated pathways are enriched in genes with higher expression in July. Gene-level expression values, however, may show individual variation within each pathway, and individual gene transcripts may show expression trends that differ from the overall pathway-level classification. Additionally, a targeted analysis of sex-related candidate genes previously characterized in *F. carica*, including *RAN1*, *AGAMOUS* homologs, VQ motif-containing proteins, and F-BOX family members (Mori et al., 2017; Wang et al., 2021), was performed to characterize differential expression patterns between male and female genotypes across the two phenological stages.

## 3 Results

### 3.1 Transcriptomic profiling reveals stage-dependent regulation of key metabolic pathways

Building upon the previous transcriptomic characterization of fig varieties (Marcotuli et al., 2020, Marcotuli et al., 2023a), an in-depth analysis of key metabolic pathways was conducted to elucidate the molecular mechanisms underlying seasonal syconium development. The previously identified 134 up-regulated and 142 down-regulated KEGG pathways (containing 4,664 and 4,947 genes, respectively) from the seasonal comparison (July vs April) across the three genotypes (Dottato, Petrelli, and Caprifig) were refined by excluding pathways not expressed or not relevant to plant metabolic systems, resulting in 124 up-regulated and 133 down-regulated plant-relevant pathways. Among these, detailed analysis focused on 15 up-regulated and 23 down-regulated pathways representing core processes in primary metabolism (photosynthesis, respiration, carbohydrate metabolism) and secondary metabolism (phenylpropanoid biosynthesis, terpenoid metabolism, flavonoid biosynthesis). For each selected pathway, gene annotations were searched against four model or closely related plant species, confirming the evolutionary conservation of these metabolic processes (**Table 1**). Among the down-regulated pathways, two central metabolic processes showed the most pronounced and consistent seasonal changes: photosynthesis-antenna proteins (map00196) and the citrate cycle/TCA cycle (map00020). Among the up-regulated pathways, four secondary pathways of particular biological interest were identified: O-antigen nucleotide sugar biosynthesis (map00541), riboflavin metabolism (map00740), glucosinolate biosynthesis (map00966), and brassinosteroid biosynthesis (map00905).

**Table 1:** Plant-specific KEGG pathways with unique genes identified among up- and down-regulated genes in *Ficus carica* varieties, with corresponding pathway details, number of genes for each pathway and the codes in related plant species: *Morus notabilis, Populus trichocarpa, Vitis vinifera, and Arabidopsis thaliana*.

Pathway			*Ficus carica*							
Metabolism category	Name	Details	UP-DOWN	Code	Ids n	KO n	*Morus notabilis*	*Populus trichocarpa*	*Vitis vinifera*	*A. thaliana*
Metabolism	Amino acid metabolism	Ala, Asp and Glu metab.	UP	map00250	2	2	mnt00250	pop00250	vvi00250	ath00250
	Amino acid metabolism	Histidine metabolism		map00340	1	1	mnt00340	pop00340	vvi00340	ath00340
	Xenobiotics biodegradation and metabolism	Chlorocyclohex. and chlorobenz. degrad.		map00361	2	1	–	–	–	–
	Xenobiotics biodegradation and metabolism	Fluorobenzoate degradation		map00364	2	1	–	–	–	–
	Glycan biosynthesis and metabolism	Other types of O-glycan biosynthesis		map00514	4	3	mnt00514	pop00514	vvi00514	ath00514
	Glycan biosynthesis and metabolism	Biosynthesis of various nucleotide sugars		map00541	3	3	mnt00541	pop00541	vvi00541	ath00541
	Xenobiotics biodegradation and metabolism	Toluene degradation		map00623	2	1	–	–	–	–
	Metabolism of terpenoids and polyketides	Monoterpenoid biosynthesis		map00902	1	1	mnt00902	pop00902	vvi00902	ath00902
	Metabolism of terpenoids and polyketides	Limonene degradation		map00903	1	1	–	–	–	–
	Metabolism of terpenoids and polyketides	Brassinosteroid biosynthesis		map00905	1	1	mnt00905	pop00905	vvi00905	ath00905
	Metabolism of other secondary metabolites	Glucosinolate biosynthesis		map00966	1	1	mnt00966	pop00966	vvi00966	ath00966
Global and overview	Global and overview maps	Biosynthesis of amino acids		map01230	12	10	mnt01230	pop01230	vvi01230	ath01230
Organismal Systems	Environmental adaptation	Circadian rhythm		map04710	2	2	–	–	–	–
	Environmental information processing	Regulation of actin cytoskeleton		map04810	3	2	–	–	–	–
	Sensory system	Motor proteins		map04814	8	6	mnt04814	pop04814	vvi04814	ath04814
Metabolism	Carbohydrate metabolism	Citrate cycle (TCA cycle)	DOWN	map00020	6	6	mnt00020	pop00020	vvi00020	ath00020
	Metabolism of other amino acids	Photosynthesis - antenna proteins		map00196	6	6	mnt00196	pop00196	vvi00196	ath00196
	Amino acid metabolism	Val, Leu and Ile biosyn.		map00290	2	2	mnt00290	pop00290	vvi00290	ath00290
	Glycan biosynthesis and metabolism	N-Glycan biosynthesis		map00510	2	2	mnt00510	pop00510	vvi00510	ath00510
	Glycan biosynthesis and metabolism	Various types of N-glycan biosynthesis		map00513	2	2	mnt00513	pop00513	vvi00513	ath00513
	Glycan biosynthesis and metabolism	Glycerophospholipid metabolism		map00564	12	10	mnt00564	pop00564	vvi00564	ath00564
	Glycan biosynthesis and metabolism	Ether lipid metabolism		map00565	4	4	mnt00565	pop00565	vvi00565	ath00565
	Glycan biosynthesis and metabolism	Linoleic acid metabolism		map00591	1	1	mnt00591	pop00591	vvi00591	ath00591
	Lipid metabolism	Styrene degradation		map00643	1	1	–	–	–	–
	Lipid metabolism	Butanoate metabolism		map00650	1	1	mnt00650	pop00650	vvi00650	ath00650
	Lipid metabolism	C5-Branched dibasic acid metabolism		map00660	1	1	mnt00660	pop00660	vvi00660	ath00660
	Lipid metabolism	Riboflavin metabolism		map00740	2	2	mnt00740	pop00740	vvi00740	ath00740
	Biosynthesis of other secondary metabolites	Isoquinoline alkaloid biosynthesis		map00950	1	1	mnt00950	pop00950	vvi00950	ath00950
	Biosynthesis of other secondary metabolites	TPP alkaloid biosynthesis		map00960	2	2	mnt00960	pop00960	vvi00960	ath00960
	Global and overview maps	Biosynthesis of unsaturated fatty acids		map01040	3	3	mnt01040	pop01040	vvi01040	ath01040
Genetic Info Proc.	Replication and repair	Fanconi anemia pathway		map03460	2	2	mnt03460	pop03460	vvi03460	ath03460
Environ. Info Proc.	Signal transduction	MAPK signaling pathway - yeast		map04011	1	1	–	–	–	–
	Signal transduction	Phosphatidylinositol signaling system		map04070	5	3	mnt04070	pop04070	vvi04070	ath04070
	Signal transduction	Phospholipase D signaling pathway		map04072	3	2	–	–	–	–
	Signaling molecules and interaction	Hormone signaling		map04081	2	1	mnt04081	pop04081	vvi04081	ath04081
Cellular Processes	Cell growth and death	Lysosome		map04142	6	5	mnt04142	pop04142	vvi04142	ath04142
	Cell growth and death	Endocytosis		map04144	11	7	mnt04144	pop04144	vvi04144	ath04144
Organismal Systems	Endocrine system	Cellular senescence		map04218	3	3	–	–	–	–

### 3.2 Seasonal regulation of photosynthetic and respiratory metabolism

**Figure 3:**
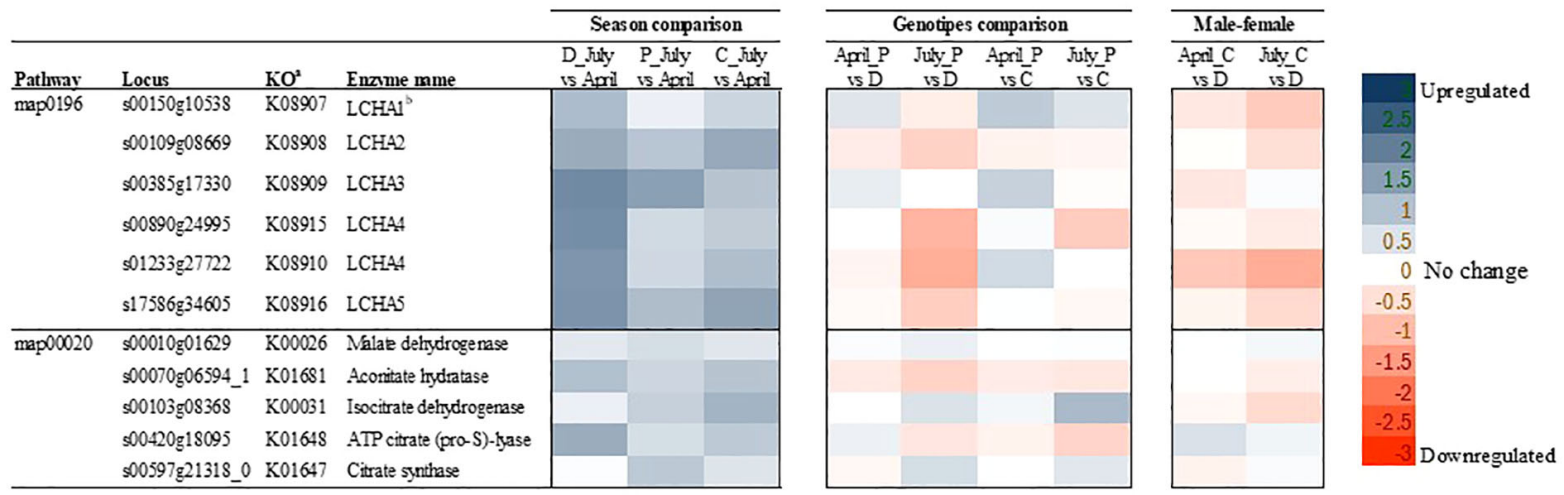
Gene-level expression changes in key metabolic pathways: TCA cycle (map00020) and Photosystem I antenna proteins (map00196). Heatmap showing log fold-change values for differentially expressed genes (DEGs) belonging to two KEGG pathways. For each gene, expression changes are displayed across three comparison groups: seasonal comparison (D_July vs April, P_July vs April, C_July vs April), genotype comparison (April_P vs D, July P vs D, April_C vs D, July_C vs D, where D = Dottato, P = Petrelli, C = Caprifig), and male- female comparison (April_C vs D, July_C vs D). Rows represent individual gene loci ordered by functional position within each pathway; columns represent pairwise contrasts. Colour scale indicates log: fold-change values ranging from -2.5 (red, downregulated) to +2.5 (blue, upregulated); white indicates no significant change. TCA cycle genes include Malate dehydrogenase (K00026), Aconitate hydratase (K01681), Isocitrate dehydrogenase (K00031), ATP citrate (pro-S)-1yase (K01648), and Citrate synthase (K01647). LHCA genes (K08907-K08916) encode subunits LHCA1-LHCA5 of the photosystem I antenna complex. ^a^KO = KEGG Orthology. ^b^LCHA1 = light-harvesting complex I chlorophyll a/b binding protein 1.

The molecular analysis revealed distinct expression patterns between April and July growth periods across the three fig genotypes (**Table 2**; **Figure 1**). For photosynthesis-antenna proteins (map00196), all six light-harvesting complex I chlorophyll a/b binding protein genes (LHCA1-5) showed substantially higher expression levels in July compared to April. The mean expression values increased from 114.89, 114.34, and 143.61 FPKM in April to 334.13, 213.69, and 306.36 FPKM in July for Dottato, Petrelli, and Caprifig, respectively, representing an approximately 1.5- to 2.9-fold increase in photosynthetic antenna protein expression during the summer period. Similarly, malate dehydrogenase, aconitate hydratase, isocitrate dehydrogenase, ATP citrate lyase, and citrate synthase genes involved in the citrate cycle (map00020; **Figure 3**) demonstrated elevated expression in July, with mean values rising from 120.96, 125.12, and 129.70 FPKM in April to 174.16, 209.40, and 265.77 FPKM in July across the three fig genotypes. Notably, isocitrate dehydrogenase (s00103g08368) exhibited the highest expression levels among TCA cycle genes, particularly in Caprifig during July (962.75 FPKM), indicating enhanced respiratory metabolism during the warmer growth period.

**Table 2:** Seasonal variation (April vs July) in three fig types (Petrelli, Dottato and Caprifig) related to pathway map00196 and map00020, with the respective loci, KO and function.

					Dottato	Petrelli	Caprifig	Dottato	Petrelli	Caprifig
Pathway	Pathway function	Locus	KOa	Function	April	April	April	July	July	July
map00196	Photosynthesis proteins	s00109g08669	K08908	LCHA1b	193.13	153.71	163.14	480.92	295.93	421.80
		s00150g10538	K08907	LCHA2	96.80	134.26	176.37	204.63	165.32	287.97
		s00385g17330	K08909	LCHA3	81.37	103.55	137.74	284.21	292.65	267.13
		s00890g24995	K08915	LCHA4	90.48	90.85	100.09	306.06	141.88	178.74
		s01233g27722	K08910	LCHA4	86.77	74.95	134.89	274.85	118.45	278.71
		s17586g34605	K08916	LCHA5	140.83	128.70	149.41	454.09	267.89	403.82
				Mean	114.89	114.34	143.61	334.13	213.69	306.36
				SD	43.89	29.57	26.43	109.09	80.63	91.38
map00020	Citrate cycle (Krebs cycle)	s00010g01629	K00026	malate dehydrogenase	60.64	66.59	63.40	81.19	99.70	87.25
		s00070g06594_1	K01681	aconitate hydratase	28.45	22.21	22.71	57.57	35.83	43.99
		s00103g08368	K00031	isocitrate dehydrogenase	361.34	371.02	417.65	445.76	640.67	962.75
		s00420g18095	K01648	ATP citrate (pro-S)-lyase	85.46	104.64	72.38	211.48	157.87	133.23
		s00597g21318_0	K01647	citrate synthase	68.90	61.13	72.39	74.79	112.95	101.62
				Mean	120.96	125.12	129.70	174.16	209.40	265.77
				SD	135.97	140.53	162.27	163.76	245.00	390.94

^a^O = KEGG Orthology. ^a^LCHA = light-harvesting complex I chlorophyll a/b binding protein 1.

For each pathway and genotype, mean values and standard deviations (SD) are reported.

To evaluate whether seasonal activation of the photosynthetic apparatus was associated with enhanced central energy metabolism, the coordination between LHCA and TCA cycle gene expression across genotypes and times of season was examined. A strong positive correlation was observed between photosynthesis-antenna proteins (map00196) and TCA cycle (map00020) pathway expression (Pearson *r* = 0.77; R²=0.59; **Figure 2**), calculated across three independent biological replicates per genotype per time point. The seasonal shift was particularly evident, with April samples clustering in the lower-left quadrant of the scatter plot (LHCA and TCA cycle expression ranging from ~110 to ~150 FPKM), while July samples occupied the upper-right region (LHCA expression from ~210 to ~340 FPKM, TCA cycle expression from ~210 to ~335 FPKM). This coordinated up-regulation during July suggests that increased photosynthetic light-harvesting capacity during the warmer growth period was accompanied by enhanced respiratory metabolism, reflecting tight coupling between carbon fixation and energy production during summer syconium development. Among genotypes, Dottato showed the highest expression levels in July for both pathways, followed by Caprifig and Petrelli.

The heatmap visualization of log fold-change values (**Figure 3**) further highlights the temporal dynamics of gene expression. In the seasonal contrast panel, all three comparisons (Dottato July vs April, Petrelli July vs April, and Caprifig July vs April) displayed highly consistent expression trends. The photosystem antenna protein genes (LHCA1-5) showed a clear and uniform increase in transcript abundance across all genotypes, with the strongest induction observed in Dottato. Caprifig and Petrelli also displayed significant gene-specific up-regulation during July, indicating more moderate seasonal modulation. A similar pattern was observed for genes associated with the TCA cycle, which were consistently up-regulated in July. However, unlike the photosystem antenna proteins, Petrelli and Caprifig displayed a more uniform and homogeneous induction across TCA-related genes, while Dottato showed greater variability in gene-specific expression responses. Conversely, the genotype comparison panel revealed a heterogeneous distribution of expression signals without a dominant directional pattern, and the male-female comparison panel showed condition-dependent expression differences. Overall, the clear and coordinated induction observed in seasonal comparisons contrasts with the more fragmented and gene-specific expression patterns detected in genotype and sex contrasts, indicating that temporal and environmental signals apply a stronger and more uniform regulatory effect on both photosynthetic and respiratory metabolism.

**Figure 4:**
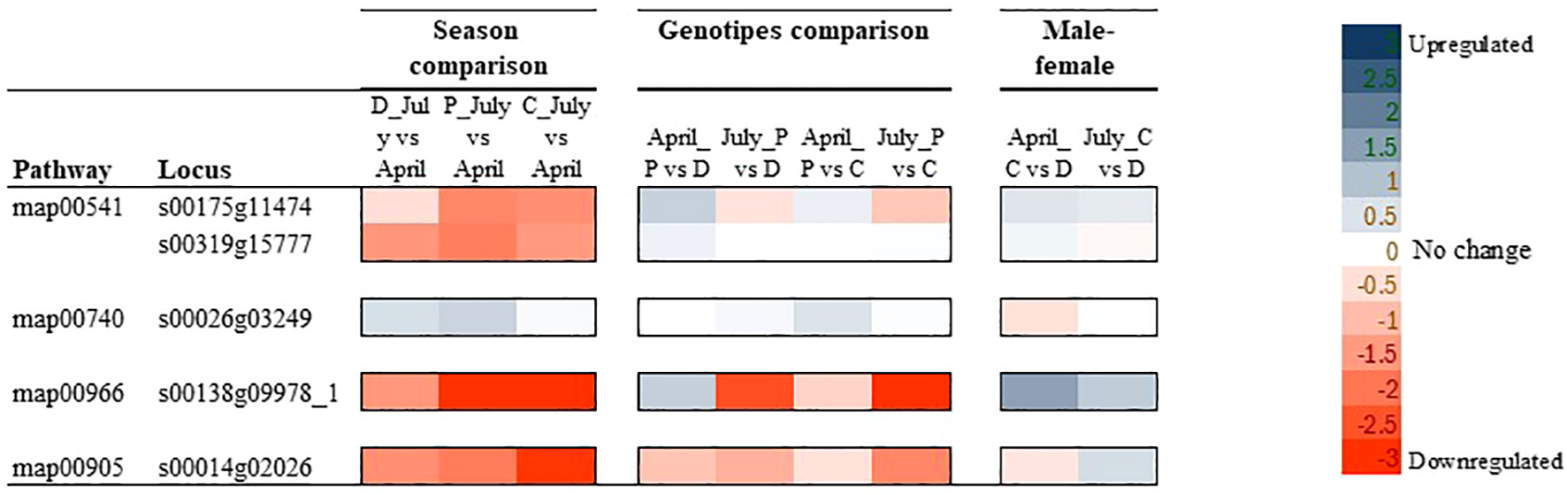
Integrated heatmap of additional seasonally modulated metabolic pathways.

Among the sex-related candidate genes additionally examined, *RAN1* and *AGAMOUS* homologs showed the most consistent seasonal downregulation, particularly in Caprifig, while *VQ* and *F-BOX genes* displayed more variable patterns across genotypes and seasons (**Figure 4**).

### 3.3 Additional seasonally modulated metabolic pathways

The expression profiles of four additional metabolic pathways are reported in **Table 3** and visualized in **Figure 4** as log_2_ fold-changes (July vs April). Glucosinolate biosynthesis (map00966), represented by N-hydroxythioamide S-β-glucosyltransferase (s00138g09978_1), showed the most pronounced seasonal regulation, with markedly higher expression in April (Dottato 14.00, Petrelli 24.30, Caprifig 8.77 FPKM) compared to July (Dottato 4.88, Petrelli 0.83, Caprifig 0.46 FPKM). Brassinosteroid biosynthesis (map00905), encoding 3-epi-6-deoxocathasterone 23-monooxygenase (s00014g02026), similarly showed higher expression in April (Dottato 110.02, Petrelli 59.11, Caprifig 78.67 FPKM) than in July (Dottato 35.48, Petrelli 15.70, Caprifig 10.46 FPKM). Biosynthesis of nucleotide sugars (map00541) was represented by two genes with distinct expression patterns: GDP-mannose 4,6-dehydratase (g00175g11474) and UDP-glucose 6-dehydrogenase (s00319g15777), both showing higher April expression across all genotypes. In contrast, riboflavin kinase (s00026g03249; map00740) was the only gene showing higher expression in July (Dottato 29.19, Petrelli 33.34, Caprifig 31.65 FPKM) than in April (Dottato 19.90, Petrelli 20.30, Caprifig 28.22 FPKM), suggesting an increased demand for riboflavin-dependent enzymatic activity during summer development.

**Table 3:** Selected metabolic pathways involved in fig seasonal development: functional annotation (pathway ID and regulation direction), cross-species conservation (*Morus notabilis, Populus trichocarpa, Vitis vinifera, Arabidopsis thaliana*), and gene expression levels (mean FPKM) across genotypes (D = Dottato, P = Petrelli, C = Caprifig) and seasons.

										D	P	C	D	P	C
Pathway	Pathway function		Morus	Populus	Vitis	Arab.	Locus	KOa	Function	April	April	April	July	July	July
map00541	Nucleotide sugars biosyn.	UP	mnt00541	pop00541	vvi00541	ath00541	s00175g11474	K01711	GDPmannose 4,6-dehydratase	64.52	109.65	79.35	45.10	32.61	25.18
							s00319g15777	K00012	UDPglucose 6-dehydrogenase	193.39	233.31	198.92	66.25	63.72	71.16
map00740	Riboflavin metabolism	DOWN	mnt00740	pop00740	vvi00740	ath00740	s00026g03249	K20884	riboflavin kinase	19.91	20.30	28.23	29.20	33.34	31.65
map00966	Glucosinolate biosyn.	UP	mnt00966	pop00966	vvi00966	ath00966	s00138g09978	K11820	N-hydroxythioamide S-β-GT	14.00	24.30	8.77	4.89	0.83	0.46
map00905	Brassinosteroid biosyn.	UP	mnt00905	pop00905	vvi00905	ath00905	s00014g02026	K12638	CYP90C1/CYP90D1	110.03	59.11	78.68	35.48	15.70	10.47

## 4 Discussion

The present study provides a refined transcriptomic perspective on the seasonal regulation of primary metabolic pathways in fig, demonstrating that temporal environmental signals induce consistent, directional transcriptional changes across genotypes that exceed the more heterogeneous and variable effects associated with genotype and sex. The seasonal transition from April to July elicited coordinated regulation of core metabolic pathways, including photosynthesis, respiration, and secondary metabolism, with remarkably uniform expression patterns across the male Caprifig and the female cultivars (Dottato and Petrelli), indicating that seasonal cues override cultivar-specific regulatory programs. Through the application of a stringent filtering strategy to previously generated RNA-seq datasets (Marcotuli et al., 2020), pathway redundancy was reduced and a biologically coherent set of genes was retained, enabling the identification of metabolic processes robustly modulated by developmental stage comparison. Cross-validation of gene annotations across multiple model and woody plant species supports the evolutionary conservation and biological reliability of the selected pathways. Although the transcriptomic patterns identified in this study were supported by stringent statistical criteria and consistent biological trends across the three fig genotypes, independent validation of selected DEGs by RT-qPCR will be necessary to further confirm the expression profiles observed.

The molecular mechanisms underlying these seasonal transcriptional changes likely involve the integration of multiple environmental signals, including temperature, photoperiod, and hormonal cues, which are known to coordinate metabolic reprogramming in perennial fruit species (Cherian et al., 2014). In fig specifically, these signals may act through regulation of carbon partitioning between source and sink tissues during the transition from breba to main crop development. Among the down-regulated pathways identified in the global KEGG filtering framework, photosynthesis-antenna proteins and the TCA cycle emerged as the most responsive to seasonal variation. Although formally identified as “down-regulated,” gene-level inspection revealed a consistent transcriptional increase from April to July for both pathways. In particular, the light-harvesting complex I chlorophyll a/b binding proteins (LHCA1-5) exhibited substantial expression increases (1.5- to 2.9-fold) from spring to summer across all three genotypes, indicating a generalized seasonal enhancement of light-harvesting capacity (Jansson, 1994; Ballottari et al., 2007; Klimmek et al., 2006). Their coordinated activation suggests that fig syconia and associated tissues may retain a residual photosynthetic capacity even at advanced developmental stages, potentially representing an adaptive response to peak summer irradiance and temperature (Ballottari et al., 2007; Klimmek et al., 2006; Vemmos et al., 2013).

Parallel to the activation of the photosynthetic antenna system, the concomitant up-regulation of key TCA cycle enzymes (isocitrate dehydrogenase, malate dehydrogenase, aconitate hydratase, ATP citrate lyase, and citrate synthase) indicates a seasonal intensification of mitochondrial respiratory activity and central carbon metabolism. The TCA cycle represents the center of aerobic respiration, supplying both metabolic energy (ATP, NADH) and carbon skeletons required for biosynthetic pathways (Sweetlove et al., 2010; Millar et al., 2011; Nunes-Nesi et al., 2013; Lancien et al., 2000; Lemaitre and Hodges, 2006). Its coordinated induction combined with light-harvesting components suggests that enhanced carbon fixation during summer is tightly coupled with increased energetic throughput to sustain the anabolic and developmental demands of syconium growth and fruit bud differentiation (brebas). This functional linkage is further supported by the strong positive correlation observed between LHCA and TCA pathway expression (*r* = 0.77). The particularly high expression of isocitrate dehydrogenase in caprifig during July (962.75 FPKM) may point to a genotype-specific amplification of respiratory flux potentially associated with differences in growth dynamics, sink strength, or reproductive biology. The increase in isocitrate dehydrogenase during the growth and ripening of caprifig, Dottato, and Petrelli syconia may indicate a climacteric peak of respiration in the receptacle, as a link between ethylene induction and respiration via activation of SlICDH1 (isocitrate dehydrogenase 1) has been reported in the climacteric fruit tomato (Gamrasni et al., 2020), suggesting that fig may also exhibit climacteric behavior at least in the receptacle where female flowers are located.

The expression profiles of secondary metabolic pathways revealed a distinct seasonal metabolic shift from defense and growth processes in spring to energy-intensive activities during summer syconium development. Glucosinolates are nitrogen- and sulfur-containing secondary metabolites that function primarily in plant defense against herbivores and pathogens (Halkier and Gershenzon, 2006; Brown et al., 2003; Mithen et al., 2010; Velasco et al., 2007). Their elevated expression in April suggests increased investment in chemical defense during early developmental stages when young tissues are most vulnerable to biotic stress, consistent with optimal defense theory (Zangerl and Rutledge, 1996). Brassinosteroids regulate diverse aspects of plant growth and development, including cell expansion, vascular differentiation, and stress responses (Zhu et al., 2013). The higher expression of 3-epi-6-deoxocathasterone 23-monooxygenase during April indicates an active growth program during early syconium formation, consistent with the role of brassinosteroids in cell elongation and division (Vriet et al., 2012; Fu et al., 2008; Symons et al., 2006) and their accumulation in floral organs (Matsuura-Tokita et al., 2025). The up-regulation of riboflavin metabolism during July represents a metabolically distinct response: riboflavin (vitamin B2) serves as a precursor for the coenzymes FAD and FMN, which are essential for numerous oxidation-reduction reactions including TCA cycle function and the electron transport chain (Bacher et al., 2000; Fischer and Bacher, 2005; Powers, 2003). The nucleotide sugar biosynthesis pathway, represented by GDP-mannose 4,6-dehydratase and UDP-glucose 6-dehydrogenase, showed higher expression in April, consistent with active cell wall biosynthesis and remodeling during early developmental stages (Reiter and Vanzin, 2001; Seifert, 2004; Sato et al., 2013; Kohlberger et al., 2018).

In contrast to the pronounced and coherent seasonal signal, genotype differences were comparatively slight and largely gene-specific. Dottato exhibited the highest expression levels for both photosynthetic and respiratory genes in July, consistent with the robust metabolic capacity required for parthenocarpic syconium development (Fernie et al., 2020; Smith et al., 2018). Petrelli showed more moderate seasonal modulation, possibly reflecting a distributed energy investment strategy adapted to sustaining two distinct fruiting periods, breba more intense and main crop very light. Caprifig displayed distinct metabolic characteristics including the highest isocitrate dehydrogenase expression in July, possibly supporting the specialized metabolic demands of pollen production and syconium development (Wang et al., 2022). Among the sex-related candidate genes examined, *RAN1* showed the most consistent seasonal downregulation from April to July across all three genotypes, with the most pronounced decrease observed in Caprifig, suggesting a seasonal repression of sex-determination pathways during summer syconium development. *AGAMOUS* homologs displayed a similar but less pronounced seasonal pattern, while *VQ* and *F-BOX* genes showed more variable and inconsistent expression patterns across genotypes and seasons (**Figure 4**).

The stronger and more consistent regulatory influence of seasonal cues compared with genotypic or sex-related factors underlines the predominant role of environmental/temporal factors in coordinating metabolic programs during fig syconium development (Cherian et al., 2014; Gambetta et al., 2020). It should be noted that, although April and July represent two ecologically relevant and contrasting phenological windows common to all three genotypes, the specific developmental stages differ among them: in April, Dottato is at early floral bud development (main crop) and bears very few brebas (or even nothing), Petrelli is actively developing breba syconia and may produce few main crop, and Caprifig is developing profichi and bears mamme; in July, Dottato is in the main crop development phase, Petrelli shows a very limited main crop, and Caprifig is developing mamme and producing mammoni. This genotype-specific developmental asynchrony should be considered when interpreting the observed transcriptional differences among genotypes. Future transcriptomic analyses based on recently available haplotype-resolved fig genome assemblies (Usai et al., 2025) may further improve transcript annotation accuracy and differential expression resolution.

The strong seasonal regulation of metabolic pathways identified in this study has several practical implications for fig cultivation and breeding programs. The coordinated up-regulation of photosynthetic and respiratory metabolism during July indicates a critical period of high metabolic demand when adequate nutrition, water availability, and optimal environmental conditions are particularly important for syconium quality and yield. The identification of genotype-specific metabolic profiles, particularly the robust metabolic capacity of Dottato and the specialized metabolism of Caprifig, highlights candidate genes and pathways that may serve as a foundation for developing molecular markers to inform breeding efforts aimed at improving parthenocarpic syconium development, stress tolerance, or adaptation to diverse environmental conditions.

## 5 Conclusions

This study indicates that fig syconium development is marked by pronounced phenological coordination of photosynthetic and respiratory metabolism, with a progressive metabolic shift from defense- and growth-related processes in spring toward more energy-intensive metabolic activities in summer. The tight connection between light-harvesting capacity and TCA cycle activity reveals a fundamental metabolic architecture supporting syconium development under changing environmental conditions. While genotypic and sex-related differences contribute to metabolic diversity among fig varieties, signals associated with phenological stage appear to exert the strongest regulatory influence on core metabolic pathways.

Future research should investigate the upstream mechanisms controling these seasonal metabolic transitions, including the roles of temperature, photoperiod, and hormonal signaling in coordinating photosynthetic and respiratory gene expression. By integrating metabolomic profiles (Marcotuli et al., 2023b), the research would provide a more complete picture of how gene expression changes translate into metabolite accumulation and syconium quality characteristics. Additionally, comparative analysis across broader germplasm collections could identify metabolic diversity to exploit in breeding new cultivars adapted to changing climatic conditions or with enhanced nutritional properties. The molecular framework established in this study provides a foundation for understanding the metabolic basis of fig syconium development and offers potential targets for crop improvement through both conventional breeding and biotechnological approaches.

## Data Availability

The datasets presented in this study can be found in online repositories. The names of the repository/repositories and accession number(s) can be found in the article/**Supplementary Material**.
