# Supplementary figures and images for: Transcriptomic analysis of three fig (Ficus carica L.) genotypes reveals metabolic reprogramming during seasonal development
Source: Front Plant Sci. 2026 Jul 2;17:1866576. doi: 10.3389/fpls.2026.1866576 (PMC13372901; doi:10.3389/fpls.2026.1866576)

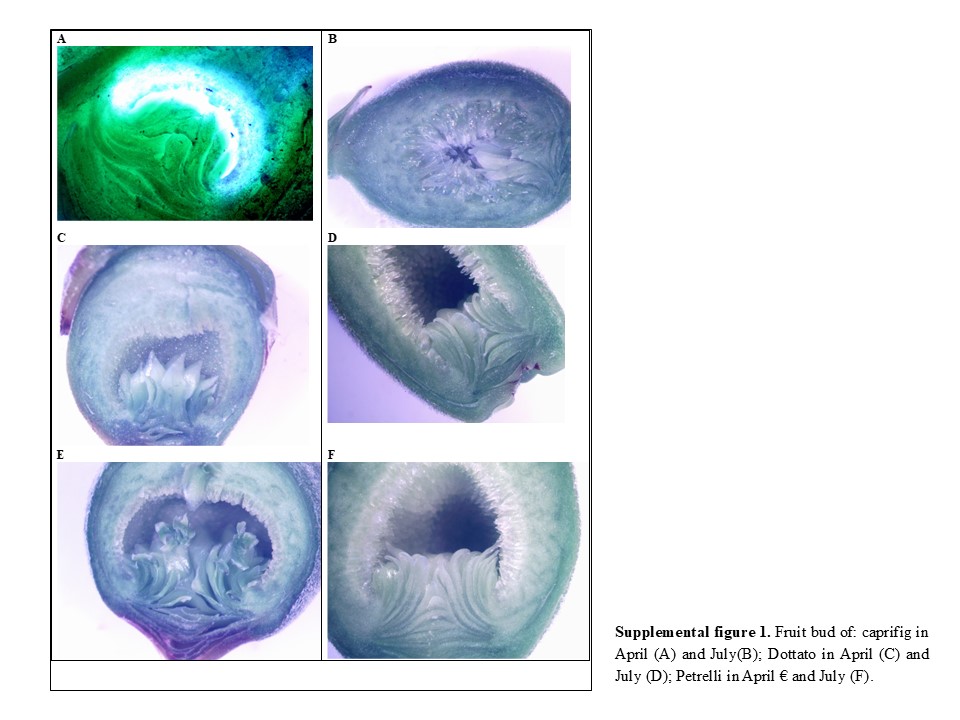

Supplement: Supplementary file 1 [file Image1.jpeg]

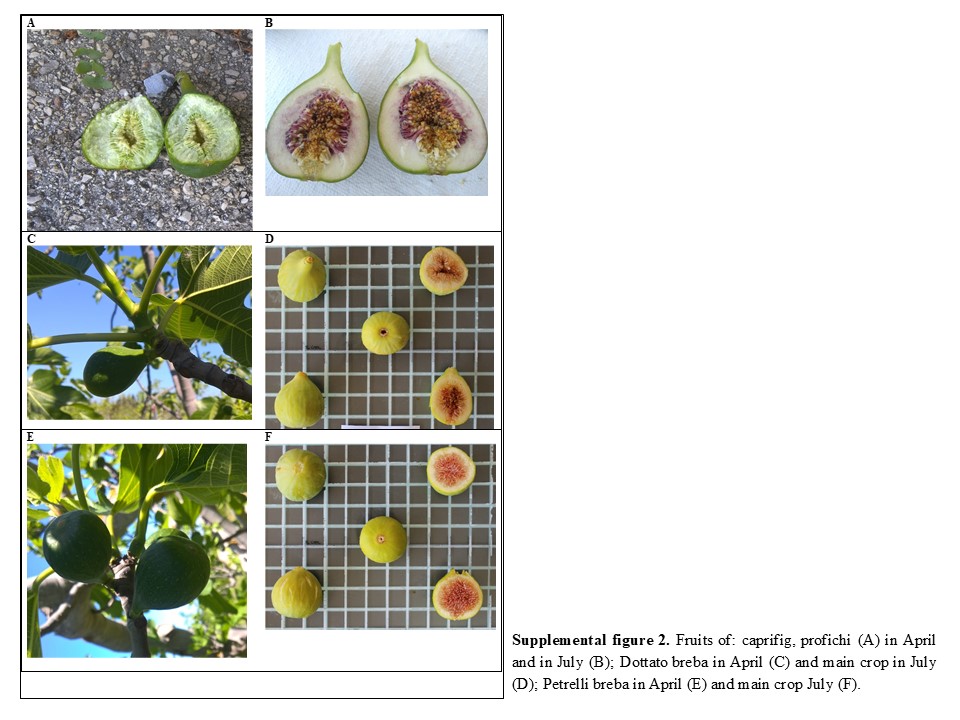

Supplement: Supplementary file 2 [file Image2.jpeg]

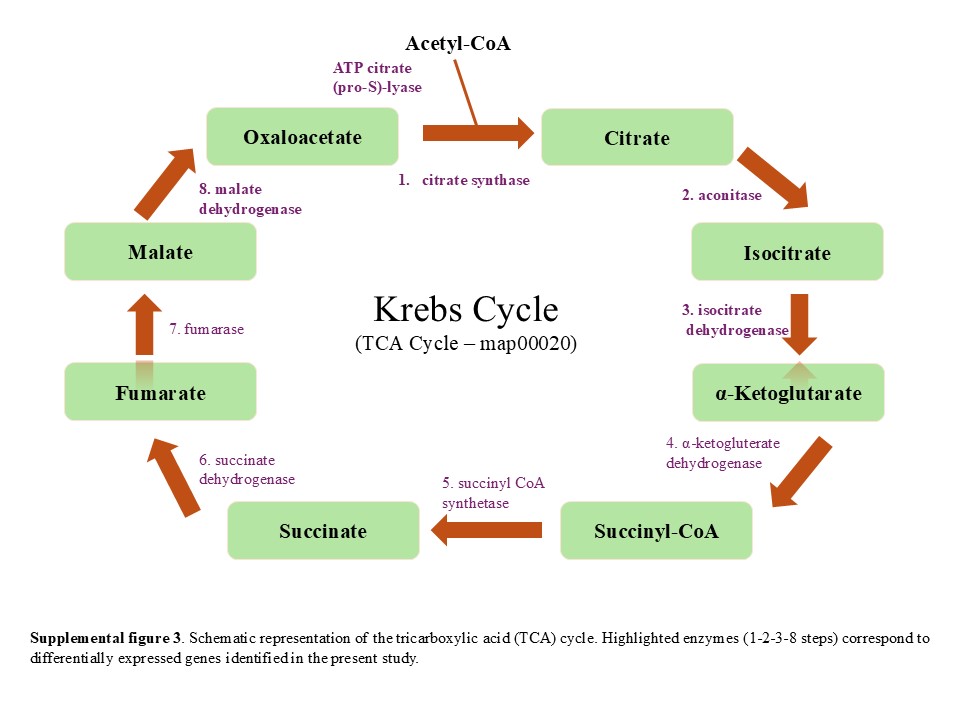

Supplement: Supplementary file 3 [file Image3.jpeg]

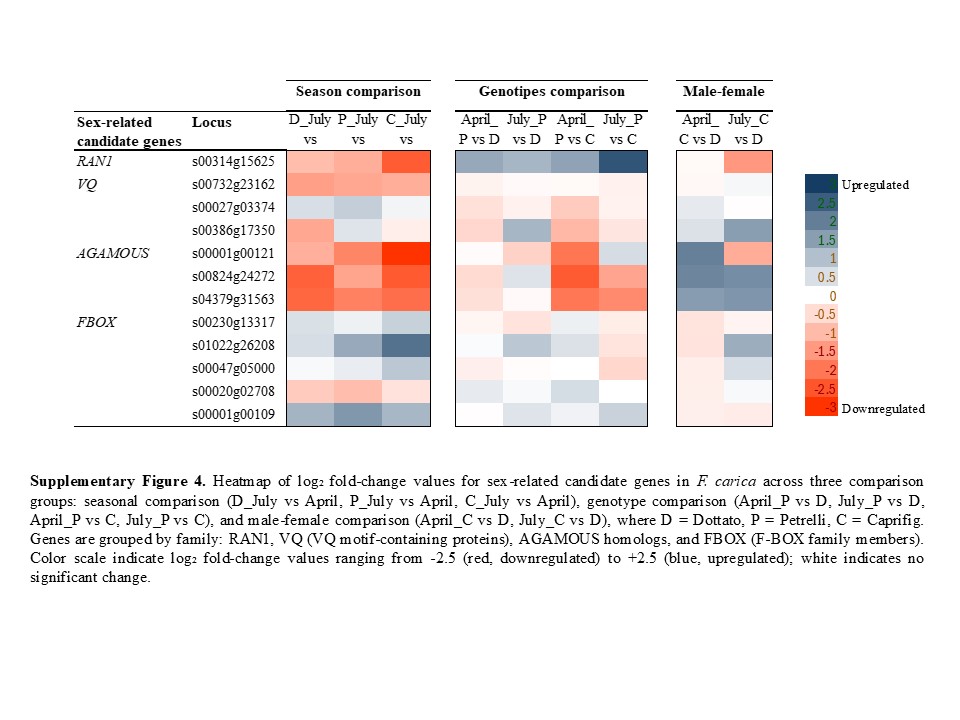

Supplement: Supplementary file 4 [file Image4.jpeg]
